# A case of the girl with ruptured bladder caused by rectal dilatation due to imperforate anus

**DOI:** 10.1111/ped.70122

**Published:** 2025-06-10

**Authors:** Nao Takami, Akinobu Taniguchi, Toshihiko Suzuki, Ryosuke Miura, Yukako Muramatsu, Yoshiaki Sato

**Affiliations:** ^1^ Division of Neonatology, Center for Maternal‐Neonatal Care, Nagoya University Hospital 65 Tsurumai‐Cho Showa‐Ku Nagoya 4668560 Aichi Japan

**Keywords:** ascites, bladder rupture, imperforate anus, neonate, surgery

Neonatal bladder rupture is rare, and most previous cases have involved boys with urinary tract obstruction due to a posterior urethral valve.^1^ We report a case involving a girl with trisomy 21 who was noted to have ascites during the fetal period and was diagnosed with a ruptured bladder after birth. Informed consent was obtained from the parents.

The 43‐year‐old mother was referred to our hospital at 36 weeks of gestation due to fetal ascites and intestinal dilation, initially suspected to be meconium peritonitis. Ultrasound revealed low amniotic fluid levels (amniotic fluid index <1 cm), bilateral Grade 2 hydronephrosis, right hydroureter, bowel dilation (Figure 1a), and marked ascites. An emergency cesarean section was performed on the same day because of decreased fetal movement. A female infant was born weighing 2400 g, with Apgar scores of 6 at 1 min and 9 at 5 min. After birth, an imperforate anus was observed. X‐ray showed bowel dilation (Figure 1b). Retrograde cystography was performed on day 1. Following lower ureterography, contrast medium was observed leaking into the intraperitoneal space (Figure 1c), raising suspicion of a ruptured bladder. No fistula was found between the rectum, vagina, and urethra. A balloon catheter was placed to decompress the bladder. Surgery was performed to repair the imperforate anus and bladder rupture. The ascites was clear and pale yellow. A fragile bladder wall was observed on the left side of the bladder body (Figure 1d). When saline was injected into the bladder through a catheter, it leaked into the intraperitoneal space. The perforation was sutured, and anoplasty was performed. Biochemical examination of the ascites showed a blood concentration of urea nitrogen of 26.7 mg/dL and a creatinine concentration of 1.54 mg/dL, indicating that the ascites was urine. Blood tests showed no hyponatremia or hyperkalemia. After the urinary catheter was removed, sufficient spontaneous urination was maintained. On ultrasound, the hydroureter disappeared and the hydronephrosis improved to Grade 1. Magnetic resonance imaging revealed no abnormalities in the spine or spinal cord. Retrograde cystography on day 23 showed no vesicoureteral reflux. Trisomy 21 was diagnosed through chromosome analysis. No ascites recurrence was observed during hospitalization, and the patient was discharged on day 40.

**FIGURE 1:**
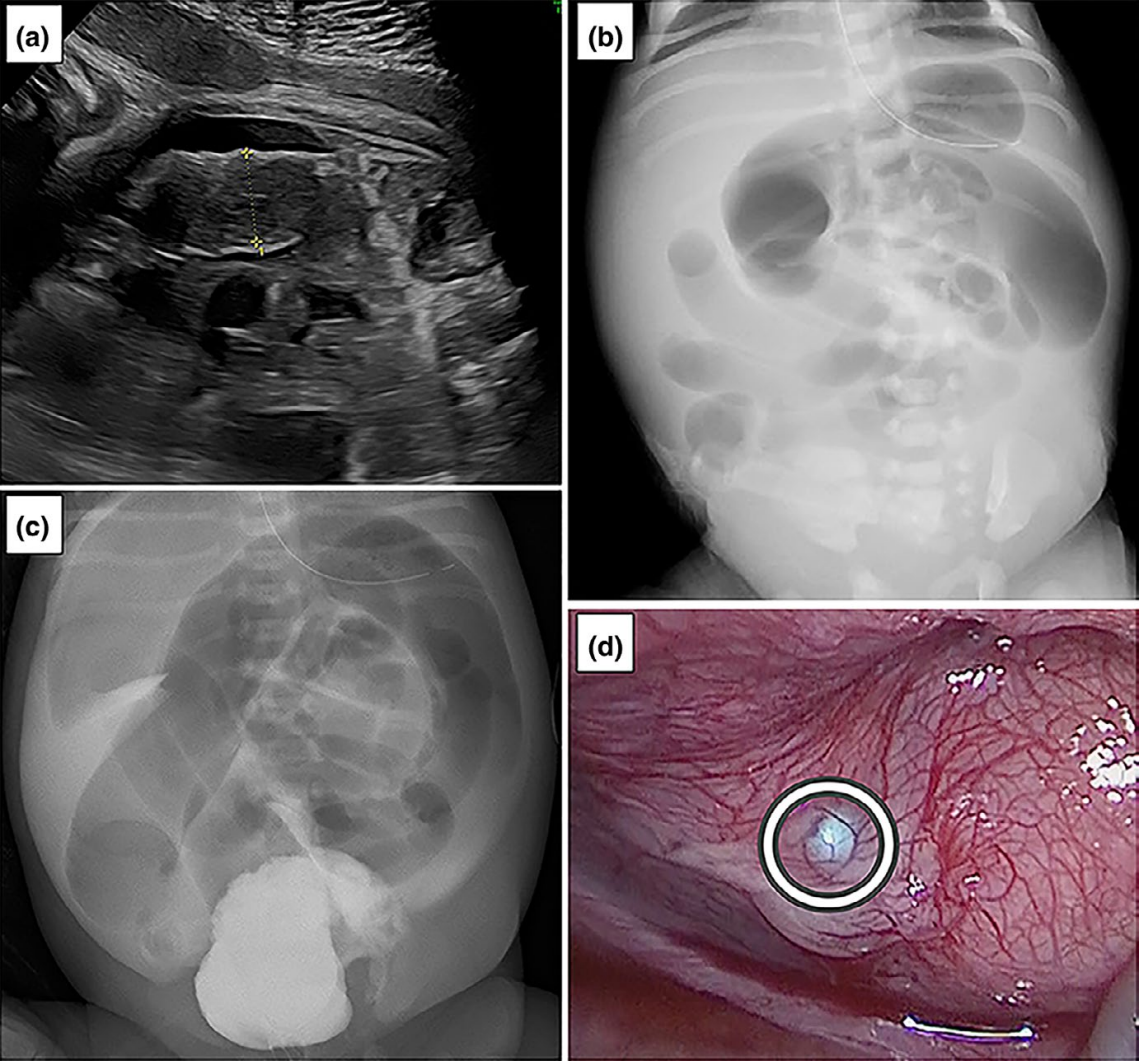
(a) Fetal ultrasound showing bowel dilation, (b) X‐ray taken after birth showing bowel dilation extending from the colon to the rectum, (c) X‐ray taken during cystography showing contrast medium leakage into the intraperitoneal space, (d) Image of the ruptured bladder.

Fetal ascites is uncommon, with 30% of cases originating from urinary causes.^2^ Neonatal bladder rupture is also very rare. There are several causes of bladder rupture, including urinary tract malformations, neurogenic bladder, and perforation by a catheter. Although various causes have been reported, most in the fetal to postnatal period are due to a posterior urethral valve; hence, most reported cases involve boys. Weller et al. reported a case of bladder rupture caused by urethral obstruction due to neuroblastoma.^3^ Chute et al. reported a bladder rupture adult case due to bowel dilatation from fecal embolism. They suggested pressure from nearby tissues caused thinning of the bladder, impairing blood flow and leading to perforation.^4^ Our case was a “covered anus complete”, in which the rectum is prone to dilatation because there is no fistula. In addition, it is difficult to diagnose an imperforate anus in a fetus, but in this case, the fetal ultrasound showed severe intestinal dilation. Based on these conditions, we thought, as in our case, pressure from severe rectal dilation caused urinary tract obstruction, leading to thinning of the bladder wall, impaired blood flow, and eventual rupture. Imaging and ascites examinations are useful for diagnosing bladder rupture. When ascites is detected, hydronephrosis and bladder wall thickness should be checked to differentiate bladder rupture. If the ascites is urine, biochemical tests will show low concentrations of protein and sodium and high blood concentrations of urea nitrogen, creatinine, and potassium. Blood tests will show similar trends because urine accumulating in the abdominal cavity acts as an auto‐dialysis agent.^5^ Severe hyponatremia and hyperkalemia sometimes occur. In our patient, the biochemical results of the ascites strongly indicated that the fluid was urine.

In summary, we report a girl with a trisomy 21 case whose bladder rupture was suspected to have been caused by urethral compression due to imperforate anus. If a patient suspected of having imperforate anus has ascites, clinicians should consider the possibility of bladder rupture due to increased pressure from imperforate anus.

## AUTHOR CONTRIBUTIONS

NT was responsible for writing the manuscript. AT supervised the whole process, including treatment. TS was responsible for treating the patient. YS, YM, and RM critically reviewed the manuscript and contributed to the conception of this report. All authors read and approved the final manuscript.

## CONFLICT OF INTEREST STATEMENT

The authors declare no conflict of interest.
